# Supplementary figures and images for: In Vivo Studies of Inoculated Plants and In Vitro Studies Utilizing Methanolic Extracts of Endophytic Streptomyces sp. Strain DBT34 Obtained from Mirabilis jalapa L. Exhibit ROS-Scavenging and Other Bioactive Properties
Source: Int J Mol Sci. 2020 Oct 6;21(19):7364. doi: 10.3390/ijms21197364 (PMC7582327; doi:10.3390/ijms21197364)

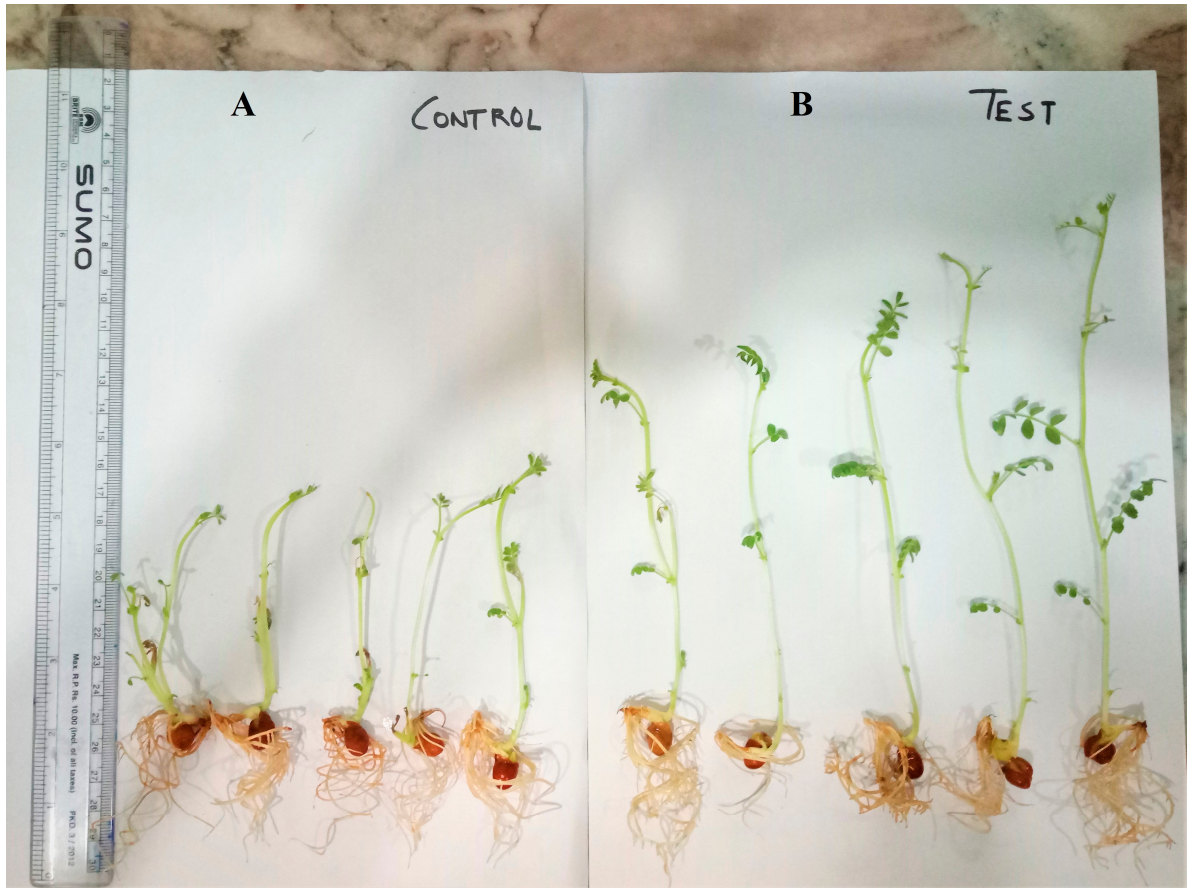

Supplement: Supplementary file 1 [file ijms-21-07364-s001.pdf]
